# Understanding the impact of dementia and age‐related vision loss on older adults’ participation

**DOI:** 10.1002/alz70858_097822

**Published:** 2025-12-24

**Authors:** Colleen McGrath

**Affiliations:** ^1^ Western University, London, ON, Canada

## Abstract

**Introduction:**

Although a dual diagnosis of dementia and age‐related vision loss (ARVL) is common in later life, there is minimal research to describe its impact on participation. As such, the aim of this scoping review was to explore the combined impact of ARVL and dementia on the participation of older adults, with a specific focus on highlighting strategies that help mitigate the impact of ARVL and dementia on participation.

**Methods:**

This study adopted a scoping review methodology based off the Arksey & O’Malley (2005) framework. An exhaustive search of 62 terms across six databases (PubMed, CINAHL, Scopus, EMBASE, Medline, and PsycINFO) was conducted. In addition, the reference lists of included articles, as well as grey literature, including conference proceedings, textbooks, and websites were searched. To be included in the review, each study had to: 1) involve older adults (65+ years old); b) be focussed on the outcome of participation; c) include a dual diagnosis of dementia and ARVL; d) be written in English and e) be available through the university's library database.

**Results:**

After a title, abstract, and full‐text screen, 13 research articles and 10 grey literature sources that met the inclusion criteria were included in the final review. Following detailed thematic analysis of the empirical and grey literature sources, four themes emerged regarding the impact of combined ARVL and dementia on the participation of older adults including: 1) Managing the pragmatic aspects of a dual diagnosis; 2) Diverse approaches to risk assessment and management; 3) Adopting a multi‐disciplinary approach to facilitate care and 4) Using compensatory strategies to facilitate participation.

**Discussion/Conclusion:**

With the growing number of older adults aging with a dual diagnosis of dementia and ARVL, it is imperative to understand the unique participation needs of this population to develop appropriate and targeted rehabilitation services. Perhaps most pressing is the need for collaborative working relationships to develop across practice areas, such that vision professionals and dementia care professionals could share best practices, knowledge, and skills to improve the quality of care, and support the participation, of older adults aging with this dual diagnosis.

